# Environmental fate and safety analysis of methoxyfenozide application to control litchi and longan pests

**DOI:** 10.1007/s11356-024-33677-0

**Published:** 2024-05-21

**Authors:** Yanping Liu, Xiaonan Wang, Siwei Wang

**Affiliations:** Key Laboratory of Green Prevention and Control On Fruits and Vegetables in South China Ministry of Agriculture and Rural Affairs, Guangdong Provincial Key Laboratory of High Technology for Plant Protection, Plant Protection Research Institute, Guangdong Academy of Agricultural Sciences, Guangzhou, 510640 People’s Republic of China

**Keywords:** Methoxyfenozide, Control effect, Environmental fate, Health risk assessment, Litchi and longan

## Abstract

Litchi and longan pests significantly affect crop yield and quality. Chemical prevention and control are very effective for production; therefore, it is crucial to study fate assessment and appropriate field efficacy before pesticide application on crops to appropriately assess the health and ecological risks linked with these agents. This study conducted Good Agricultural Practice (GAP) field trials and laboratory experiments to elucidate the dissipation, terminal residues, and efficacy of methoxyfenozide on litchi and longan in six locations throughout China. To detect methoxyfenozide residues on litchi and longan, a QuEChERS/UPLC-MS/MS-based method was designed. The initial methoxyfenozide levels in litchi and longan ranged from 2.21–2.86 to 0.83–0.95 mg kg^−1^ and indicated half-lives of 5.1–5.3 and 5.3–5.7 days, respectively. After 7 days of foliage treatment, the concentrations of terminal methoxyfenozide residue were 0.78–2.61 and 0.02–1.01 mg kg^−1^, which were less than the established maximum residue limit for methoxyfenozide in litchi and longan. The chronic (acceptable daily intake = 0.0055–0.0331%) dietary intake risk analysis for methoxyfenozide in longan and litchi indicated acceptable concentrations of terminal residue for the general population. Methoxyfenozide in litchi and longan was readily degraded in first-order kinetics models, the degradation rate on longan was higher than that on litchi, and their dietary risks were negligible to consumers. Two hundred forty grams per liter of methoxyfenozide suspension concentrate (SC) represents a highly efficacious insecticidal dose to control litchi and longan pests and indicates a significant application potential as it is rapidly degraded and linked with reduced post-treatment residue levels.

**Supplementary Information:**

The online version contains supplementary material available at 10.1007/s11356-024-33677-0.

## Introduction

Insecticides are commonly used to kill pests that have a harmful impact on crop growth, thereby improving the quality and yield of various important agricultural products (Liu et al. 2021). Diazide insecticides are the ecdysone agonists of insect growth regulators that are used as third-generation environmentally friendly insecticides because of their high selectivity, low toxicity, and environmental safety (Xu and Li. 2015). Diazide insecticides mainly simulate the physiological and biochemical effects of ecdysone, causing larvae to enter the molting stage early, which are unable to complete the molting process normally and die. Since the 1990s, China has been using pesticides annually over an area of 5.8 billion acres, recovering over 58 and 1.5 million tons of grain and cotton losses, respectively, playing a crucial role in protecting human health and food security (Hu 2005). For many years, chemical control has remained an indispensable and important tool to control pests in integrated pest management (Liu et al. 2022). Methoxyfenozide acts as a stomach poison that has contact toxicity, ovicidal effects, and good control effects against pests such as thrips, striped armyworms, cotton bollworms, cabbage worms, beet armyworms, and leaf roller moths (Wu et al. 2022). Hence, methoxyfenozide has been rapidly promoted worldwide. However, some literature reports that methoxyfenozide is toxic for bees and aquatic organisms and, therefore, may pose a potential risk to human health as well (Fisher et al. 2018; Chen et al. 2021). Currently, many countries are carefully monitoring methoxyfenozide, adopting stricter standards for its residue limits, and proposing plans to limit its use (Sun et al. 2019a, b). The possible risks linked with methoxyfenozide application require careful monitoring and remain a public concern.

Both litchi (*Litchi chinensis* Sonn.) and longan* (Dimocarpus longana* Lour.) are important tropical plants that belong to the Sapindaceae family, are native to China, and have high economic benefits. Litchi has therapeutic and health benefits in all organs and has the potential for comprehensive development and utilization (Wang et al. 2009). Its pulp contains a large amount of organic acids, amino acids, soluble sugars, vitamins, and trace elements (USAD n.d.). Furthermore, its peel contains a large amount of antioxidants and phenolic compounds (Sun 2006; Li and Jiang 2007; Lin et al. 1988), whereas its seeds have antioxidant and immune-enhancing effects (Pan et al. 1999; Xiao et al. 2007). Longan is an agricultural product that serves as medicine and food. Traditional Chinese medical books have reported longan’s physiological function including immune regulation, antioxidant, and anti-tumor (Hao et al. 2021). Longan contains lipids, dietary fiber, proteins, phenols, sugars, volatile flavor compounds, vitamins, and trace elements (Rangkadilok et al. 2007). *Conopomorpha sinensis* Bradley, *Thalassodes immissaria* Walker, and *Tessaratoma papillosa* Drary are among the most serious pests that affect litchi and longan during their growth and post-harvest storage periods. They infect and eat inflorescences, young shoots, leaves, and fruits. The eaten young shoot is unable to form flowers and fruits, resulting in significant economic loss to growers (Li et al. 2018; Yao et al. 2021; Chen et al. 2020). It has been observed that methoxyfenozide protects peach, rice, and cotton from *Cnaphalocrocis medinalis*, *Carposina sasakii*, and *Helicoverpa armigera* (Wang et al. 2021; Li et al. 2022; Zhang et al. 2021). However, studies examining the dissipation features of methoxyfenozide against the pests that attack litchi and longan are still lacking.

Pesticide residue detection is generally divided into two major steps: sample pretreatment and instrumental analysis. Sample pre-treatment primarily comprises the following steps: sample processing, extraction, purification, and concentration. Instrument analysis mainly includes chromatography and spectroscopy, which can be divided into qualitative and quantitative analyses. High-performance liquid chromatography-tandem mass spectrometry (HPLC–MS/MS) techniques can effectively detect pesticide residues from various food materials because of their selectivity, power of approach, and high resolution. Therefore, HPLC–MS/MS methods are among the most efficient techniques for assessing ultra-trace level pesticide residues (Ucles et al. 2014; Pico et al. 2017). Rapid preprocessing methods and highly sensitive instrument analysis help to accurately detect pesticide residue levels in agricultural products, which is of great significance for precise pest control.

This research aims to (1) elucidate the dissipation features and distribution rules of methoxyfenozide residue levels on litchi and longan, (2) explore the factors influencing the methoxyfenozide degradation in litchi and longan, and (3) assess the associated dietary risks with this pesticide based on the acceptable daily intake (*ADI*%) systems.

## Material and methods

### Reagents and materials

The pure acetonitrile (MeCN) was acquired chromatographically from Fisher (PA, USA). Standard methoxyfenozide (98.0% purity) was obtained from AccuStandard.Inc (Yale, USA). Sinopharm Chemical Reagents Co., Ltd (Shanghai, China) provided analytical grade sodium chloride (NaCl) and anhydrous magnesium sulfate (anhydrous MgSO_4_). Graphitized carbon black (GCB) and primary, secondary amine (PSA) sorbent were provided by Agela Technologies Inc. (Tianjin, China). Formic acid (HPLC-grade) was provided by the Aladdin Industrial Corporation (Shanghai, China). Dow AgroSciences Co., Ltd provided 240 g L^−1^ methoxyfenozide SC.

To prepare ultrapure water, a Milli-Q Integral Water Purification System (Millipore Corporation; MA, USA) with a 13 mm × 0.22 μm filter membrane (Ameritech Science and Technology Ltd., Chicago, IL, USA) was used. A refrigerated high-speed centrifuge (model GTR22-1) was acquired from the Beili Medicine Centrifuge Factory (Beijing, China).

### Sample preparation

Using 20 mL of MeCN, a mashed sample (5.0 g) was extracted, shaken for 2 min, and then mixed with NaCl (1 g) and anhydrous MgSO_4_ (4 g). After another 1 min of mixing, the sample was kept on a horizontal oscillator for 10 min, then subjected to centrifugation at 5204 × g for 5 min. Subsequently, to facilitate purification, the supernatant (2 mL) was placed in a 5-mL tube containing PSA (100 mg), GCB (5 mg), and anhydrous MgSO_4_ (150 mg). After 1 min of shaking, the mixture was again centrifuged for 5 min at 5204 × g to collect supernatant, which was then filtered via a 0.22-μm nylon syringe filter and added in an autosampler vial for HPLC–MS/MS analyses.

### Instrument settings

Methoxyfenozide was separated using a Shimadzu LC-20A HPLC system with an Agilent XDB- C18 column (Dim: 50 mm × 4.6 mm, 1.8-µm particle size) and a 35 °C column oven. The mobile phase included A (0.5 mmol ammonium acetate aqueous containing 0.1% formic acid) and B (MeCN) in *v/v* = 25/75. The constant flow rate was 0.3 mL/min, and the gradient setting was as follows: 0 min = 80% A, 5.0 min = 40% A, 9.0 min = 15% A, 15 min = 5% A (keep 1.0 min), and 16.5 min = 40% A. In the autosampler, 1 μL of the sample was administered. The equilibrium and separation were achieved in 2.6 min.

For sample analysis, positive ion mode electrospray ionization (ESI^+^) attached to a triple quadrupole mass spectrometer (Shimadzu 8045; Kyoto, Japan) was used, and the instrument was set accordingly: heating block temperature = 400 °C, desolvation line (DL) = 250 °C, and oven temperature = 350 °C. Nitrogen was utilized as the nebulizer and collision gas, and methoxyfenozide was analyzed by multiple reaction monitoring. The parent ion = 369.20 m/z and the daughter or quantitative ion = 313.10 m/z and 149.10 m/z, and the collision energy (CE) was 10 eV and 25 eV, respectively.

### Methodological validation

Limit of quantification (LOQ), linear equation, and recovery rate data were utilized to elucidate the reliability and accuracy of the established protocol. Stock solutions of concentration 1000 mg L^−1^ were prepared for methoxyfenozide in acetonitrile and stored at − 20 °C. Following this, fresh working standards were prepared for calibration and fortification via dilution of stock solution. The solvent calibration solutions were prepared in acetonitrile. The matrix-matched solutions were prepared by combining blank extracts from each matrix with the stocks. All the prepared solutions were stored at 4 °C before use. The concentrations of solvent standard curves were 0.0005, 0.001, 0.005, 0.01, 0.02, 0.05, 0.1, 0.2, and 0.5 mg L^−1^, respectively. The concentrations of matrix-matched standard solutions were 0.001, 0.005, 0.01, 0.05, 0.1, and 0.5 mg L^−1^, respectively. Each of the above concentrations was all repeated three times.The recovery rate was elucidated with the help of pesticide-treated blank food samples at the concentrations of 0.001, 0.01, and 0.1 mg kg^−1^, with 5 replicates/concentration. The whole litchi and pulp and the whole longan and pulp were all used for the recovery test. For methodological precision assessment, relative standard deviations (RSDs) were employed. Moreover, each compound’s LOQ was considered the minimum assessed spiked level in the selected matrix (SANTE/11813/2017).

### Field trial tests

**Fig. 1:**
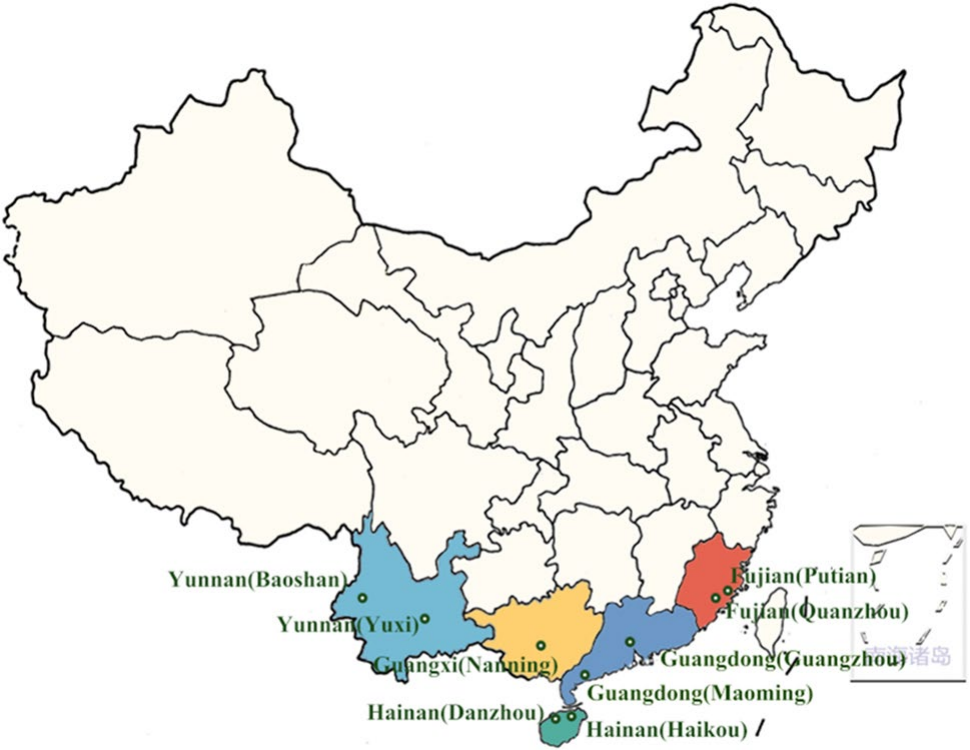
Geographical locations and final residues of the sampling sites of lychee samples in China

Open-field analyses were conducted in six litchi and longan-producing regions of China, including Guangxi (Nanning, E108.3, N22.8), Guangdong (Guangzhou, E112.57, N22.26; Maoming, E110.91, N21.6), Hainan (Haikou, E110.10, N19.32; Danzhou, E108.56, N19.11), Yunnan (Baoshan, E99.10, N25.08; Yuxi, E102.32, N24.2), and Fujian (Quanzhou, E118.37, N24.54; Putian, E119.06, N25.22) as per the Guidelines on Pesticide Residue Trials (NY/T 788–2018) issued by the Ministry of Agriculture and Rural Affairs of the People’s Republic of China (Fig. 1). For these trials, a 240 g L^−1^ methoxyfenozide suspension concentrate (SC) was sprayed onto longan and litchi at 96–144 and 144 mg a.i. kg^−1^ doses to elucidate its terminal residue behaviors and dissipation, respectively. Pre-harvest intervals were 5, 7, and 10 days, with 7 days between applications. Three replicate plots, each containing four litchi or longan trees and well-defined buffer areas to separate individual plots, were selected (Figure S1). Previous applications of other pesticides are shown in Table S1.

At least 2 kg of normally growing insects and disease-free litchi or longan samples were collected from different parts to perform sample preprocessing.

Litchi or longan whole fruit sample preparation: the impurities and tail fibers attached to the surface of the Litchi fruit were removed with a dry brush, and the fruit stem was cut off. After weighing 1/2 of the samples, they were cut into small pieces, mixed well, and divided into two parts using the quartering method. Then, two samples of 150 g were stored at − 20 ℃ for further analysis.

Litchi or longan pulp sample preparation: the remaining half of the residue samples from the field were weighed. Then their skin was peeled, the pulp was taken out, cut into small pieces, mixed well, and divided into four parts. Then, two samples of 150 g were stored at − 20 ℃ for further analysis. The residues of whole litchi and longan samples were determined during the residual dynamic trials, and the whole litchi and pulp and the whole longan and pulp samples were used in the terminal experiments.

### Dietary intake risk assessment

Generally, to elucidate the possibility of dietary intake-related chronic or acute harm to consumer’s health, *ADI%* values are employed (Feng et al. 2022; Wang et al. 2022). The *ADI%* values were assessed based on the results of the residue trial via the following formula:

$$ADI\mathrm{\%}=(STMR\times F)/(ADI\times bw)\times 100\mathrm{\%}$$

where *STMR* (mg kg^−1^) is the median pesticide residue levels calculated for litchi and longan, *bw* (kg) is the body weight, and *F* (kg) is the average daily fruit intake in China. Table S2 depicts the fruit intake and average body weight values for Chinese consumers of different sexes and ages.

The *ADI%* analyses assessed the potential risks linked with methoxyfenozide, with an *ADI%* > 1 similarly indicating the adverse effects risk related to the prolonged dietary intake.

## Results and discussion

### Methodological validation

**Fig. 2:**
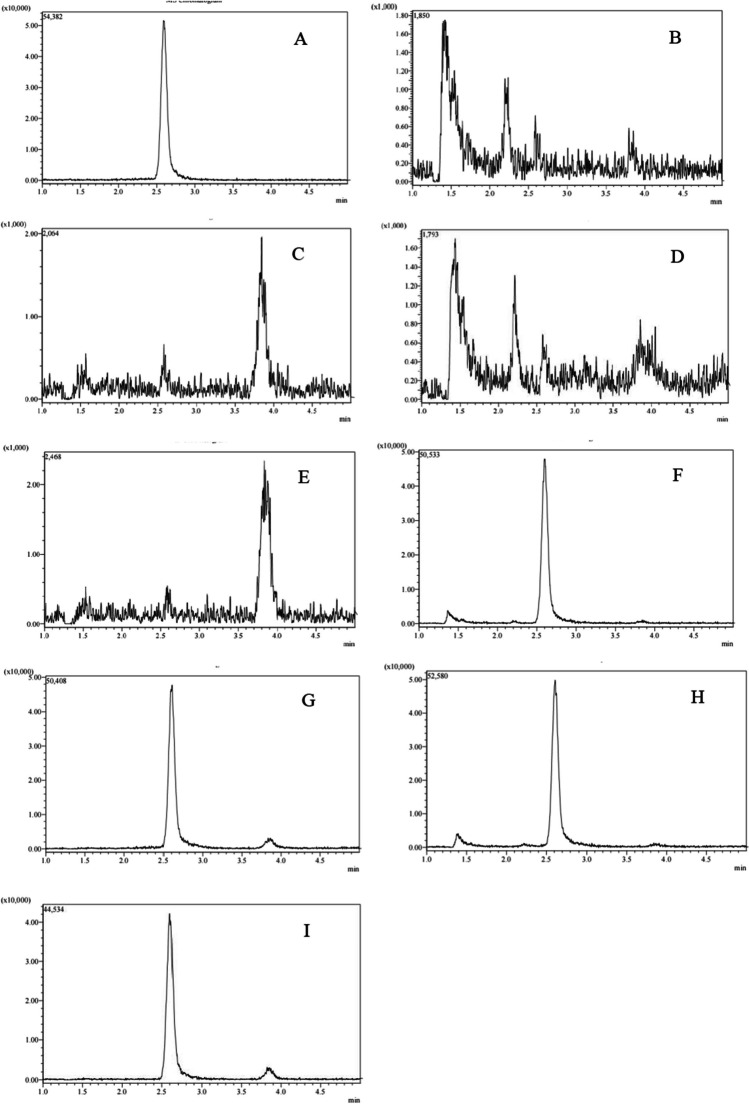
Representative UPLC-MS/MS chromatograms of the methoxyfenozide standard (1 μg L^−1^, **A**), blank whole litchi sample (**B**) and pulp (**C**), whole longan (**D**) and pulp (**E**), whole litchi (**F**) and pulp (**G**), whole longan (**H**) and pulp (**I**) spiked with methoxyfenozide (1 μg kg.^−1^)

Matrix effects can cause inaccurate quantification, which affects the sensitivity, precision, and accuracy of HPLC–MS/MS assays. To resolve these issues, this research used matrix-matched standards. Furthermore, the RSDs, LODs, recovery rates, ME, and LOQs for methoxyfenozide on litchi and longan were assessed (Fig. 2, Table 1), and the calibration curves were generated on the basis of the ratio of peak area to matrix-matched standard concentrations in the 0.0005–0.5 mg kg^−1^ range. Litchi and longan indicated average recovery rates of 83–97% and 85–95%, with RSD_a_ of 3.8–7.4% and 5.4–8.5%, and RSD_r_ of 5.2–10.6% and 6.3–11.7%, respectively, indicating satisfactory precision and accuracy. These data were similar to a target mean recovery of 70–120% and an associated RSD of ≤ 20%. Additionally, the LOQ values for methoxyfenozide on litchi and longan were 0.0005–0.001 mg kg^−1^, whereas the LODs were 0.00001–0.0003 mg kg^−1^, based on the concentrations indicating a signal-to-noise ratio of 3.

**Table 1:** Average recovery and RSDs of methoxyfenozide in litchi and longan (*n* = 5)

Matrix	Calibration curve	*R*^2^	Curve concentration range (μg L^−1^)	ME^a^ (%)	Spiked level^a^ (μg kg^−1^)	Average recovery^b^ (%)	RSD_a_^c^ (%)	RSD_r_^d^ (%)	LOQ (μg kg^−1^)	LOD (μg kg^−1^)
Solvent	*y* = 5E + 07*x* + 124352.84	0.9996	0.5–500	-	-	-	-	-	-	-
Litchi pulp	*y* = 5E + 07*x* + 199852	0.9994	0.5–500	2.51	1 10 100	83 93 97	3.9 7.4 4.7	5.2 10.6 7.1	0.5	0.01
Whole litchi	*y* = 4E + 07*x* + 184866	0.9984	0.5–500	19.8	1 10 100	87 90 85	4.3 5.6 3.8	6.4 8.8 6.1	1	0.3
Longan pulp	*y* = 5E + 07*x* + 206547	0.9996	0.5–500	2.33	1 10 100	85 91 95	8.5 5.7 5.4	11.2 9.4 6.3	0.5	0.01
Whole longan	*y* = 4E + 07*x* + 147082	0.9993	0.5–500	11.2	1 10 100	89 87 92	6.3 7.7 8.6	8.9 10.0 11.7	1	0.3

^a^The standard fungicide was spiked before the sample grinding

^b^The recovery was calculated by the formula: Recovery = *C*_*d*_* / C*_*s*_ × 100%, where *C*_*d*_ represents the detected concentration and *C*_*s*_ represents the spiked concentration. Results were expressed as mean ± standard deviation (SD) with 95% confifidence intervals

^c^Mean intra-day precisions (RSD_a_) (*n* = 5)

^d^Mean inter-day precisions (RSD_r_) (*n* = 15)

ME (Matrix effect) was calculated by the equation: ME = (slope of the matrix-matched standard/slope of the solvent standard − 1) × 100%. An ME with a negative and positive value means that the pesticide response is suppressed and enhanced. It is generally believed that when:│ME│ ≤ 20%, the matrix does not exist; 20% ≤ │ME│ ≤ 50%, it indicates a medium matrix effect; and when│ME│ ≥ 50%, it indicates a strong matrix effect

LOQ means the Limit of Quantitation, the lowest spike level

LOD means the limit of detection

### Characterization of dissipation behavior

**Fig. 3:**
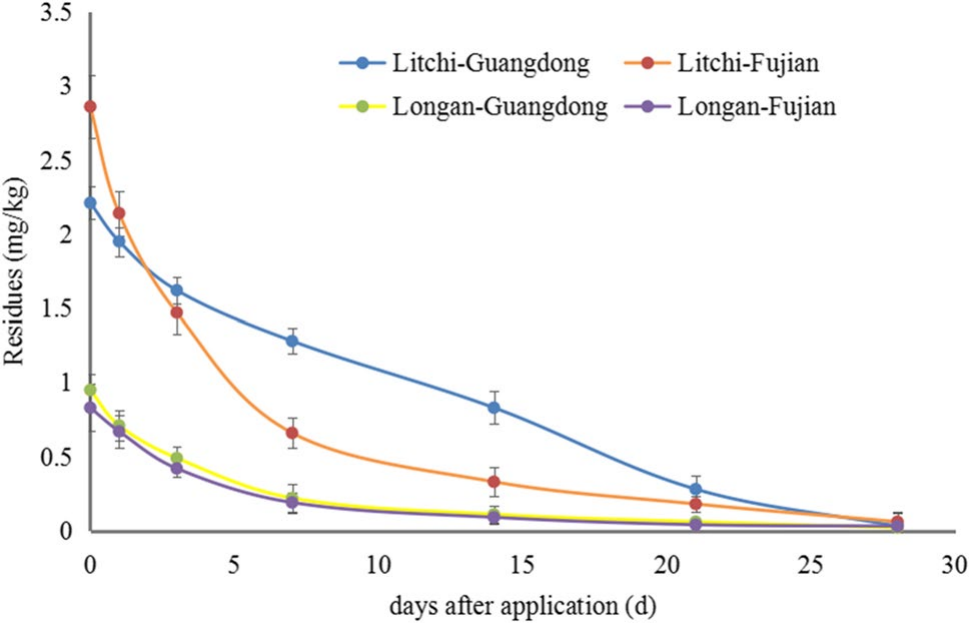
Dissipation curve of methoxyfenozide on litchi and longan

**Fig. 4:**
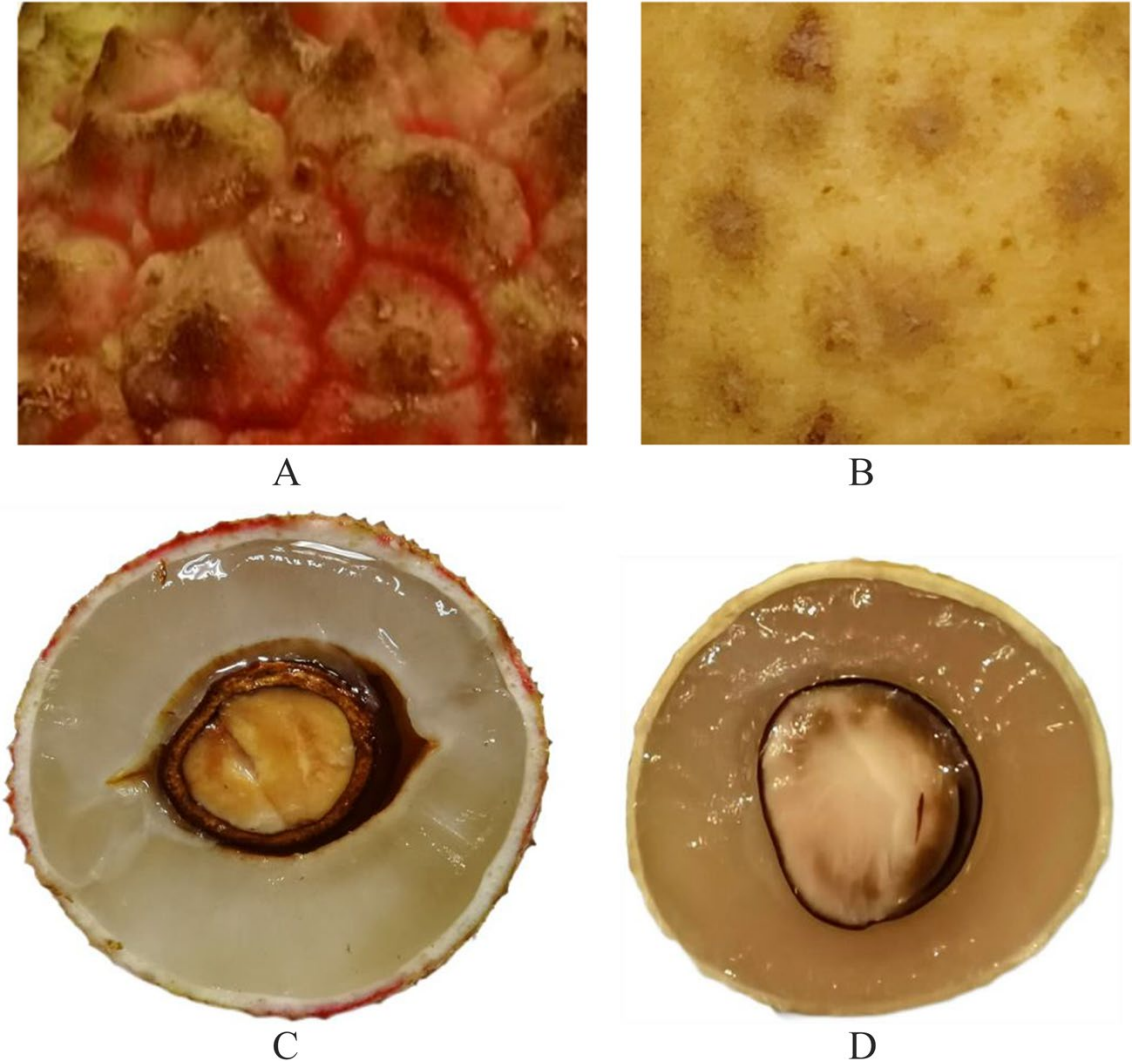
The exocarp pictures of litchi (**A**) and longan (**B**) (scale 1:10), and a cross-section of the exocarp of litchi (**A**) and longan (**B**)

The methoxyfenozide dissipation on litchi and longan followed first-order reaction kinetics *C*_*t*_ = *C*_0_ exp (-*kt*). The initial methoxyfenozide concentrations in litchi and longan samples were 2.21–2.86 and 0.83–0.95 mg kg^−1^ with half-lives of 5.13–5.33 and 5.33–5.73 days, respectively (Fig. 3). On day 21st, the dissipation rates ranged from 87 to 94% in litchi and 94–95% in longan. There is a significant difference in the initial concentrations and degradation rate of methoxyfenozide on litchi and longan. The literature indicates that many variables, such as precipitation, temperature, crop variety, and other factors, can influence the residual dissipation rates. Since both litchi and longan are Sapindaceae plants, their growth characteristics are similar. Due to the use of pesticides in the middle and later stages of fruit growth, the structural characteristics of the fruit could significantly affect the pesticide residue levels. The pericarp of litchi comprises three layers. The outer layer is composed of many hemispherical protrusions and wax coating on the surface of protrusions. The middle layer is composed of barrier and sponge tissues, which are the main part of the pericarp. The inner layer comprises several layers of small cells in the form of a very thin film tissue (Sun et al. 2019a, b; Hu 2005; Pan and Xie 1996; Zhu 2011). The longan peel can also be divided into three layers: exocarp, mesocarp, and endocarp. The exocarp and mesocarp are difficult to separate, while the endocarp and mesocarp are easily separated. The pericortex of the exocarp is composed of several stacked flat cells and is thin. Furthermore, the corky layer covering the surface of the pericortex is thin and discontinuous (Lin et al. 2002). Figure 4 indicates the exocarp pictures of litchi and longan. The surface of the exocarp on litchi is composed of many hemispherical protrusions, with a strip-like stratum corneum or wax coating on the surface. Litchi’s surface structure makes pesticide easy to retain and less prone to loss. However, longan’s outer surface is relatively smooth, and the turtle-shaped nodules are not obvious, which causes poor retention of pesticides and makes them more prone to loss. During the application period, the average temperature and rainfall in litchi and longan-producing areas were similar (Table S3); therefore, their surface structure characteristics may be the most important influencing factors. The vapor pressure, partition coefficient, and water solubility of methoxyfenozide were < 1.33 × 10^−5^ Pa, log Pow 3.72 ± 0.04 at 24.7 ± 1.4 ℃ (pH 7), and 3.3 mg/L at 20 ± 0.5 ℃ (pH 7), and 3.3 mg/L methoxyfenozide, 2003). The methoxyfenozide dissipation rates of litchi and longan were slower than those previously reported for pak choi (initial concentrations = 2.8 mg kg^−1^, half-life = 3.9 days) (Tang et al. 2021), spinach (initial concentrations = 1.2–3.8 mg kg^−1^, half-life = 1.4–3.0 days) (Feng et al. 2022), Chinese cabbage (initial concentrations = 1.12–1.33 mg kg^−1^, half-life = 1.2 days) (Wang et al. 2022), Chinese broccoli (initial concentrations = 0.27–1.73 mg kg^−1^, half-life = 1.0–5.1 days) (Bi et al. 2021), and cauliflower (initial concentrations = 2.5–3.5 mg kg^−1^, half-life = 0.14–4.38 days) (Sun et al. 2019a, b). The above reports include residue levels observed in vegetables and pesticide degradation during harvesting is generally faster due to the short growth cycle of vegetables. Hence, crop types, growth dilution rate, crop epidermal structure, and climatic conditions may be the main cause of pesticide degradation rate.

### Terminal residue assessment

**Fig. 5:**
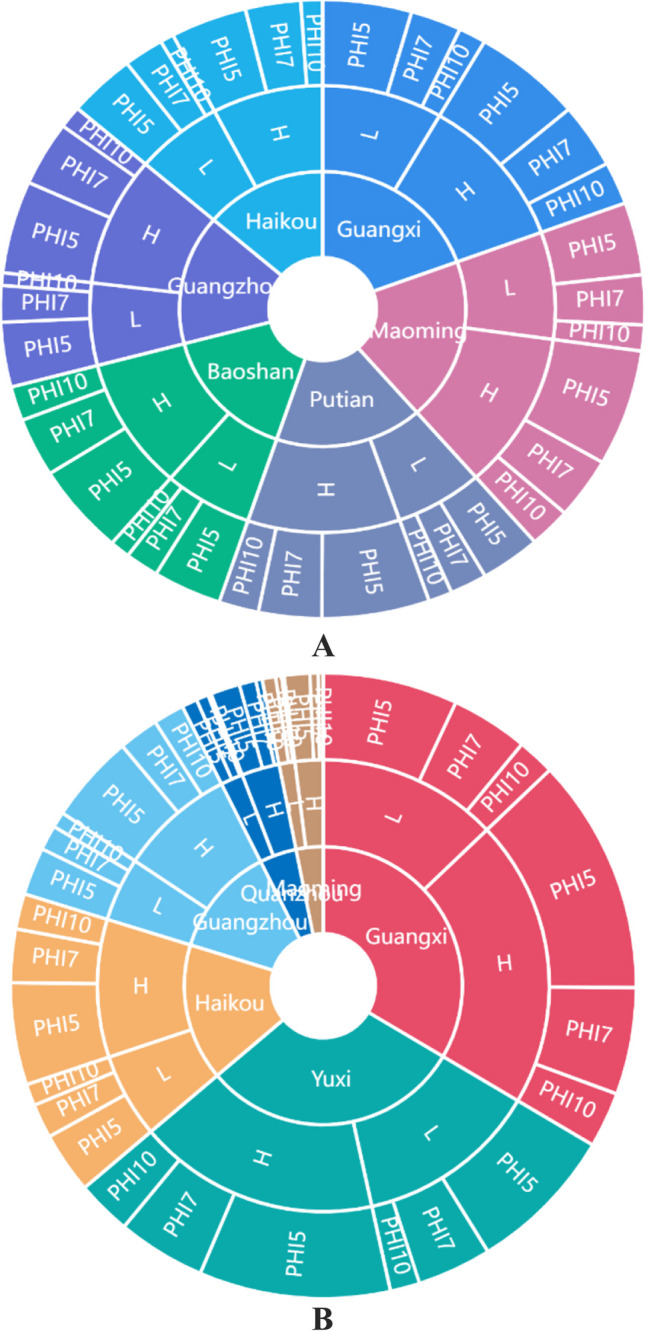
Terminal residue data of methoxyfenozide in litchi (**A**) and longan (**B**) at six locations in China

Subsequently, the terminal methoxyfenozide residues on litchi and longan at 5, 7, and 10 days after its two and three sprays (96 and 144 mg a.i. kg^−1^) were assessed in six selected fields (Fig. 5). Respective levels of whole litchi methoxyfenozide residue at the 5-, 7-, and 10-day harvest intervals were 1.22–4.88, 0.78–2.65, and 0.21–1.85 mg kg^−1^, respectively, whereas that of longan residue were 0.10–2.39, 0.04–1.01, and 0.01–0.48 mg kg^−1^, respectively. The litchi and longan pulp residues were all below LOQ at different application dosages, pre-harvest intervals (PHIs), and times. Table 2 shows the data indicating that the litchi and longan methoxyfenozide residue levels were reduced with prolonged harvest timing and enhanced with an increase in the number of applications and dosage. Moreover, no substantial differences were observed in the levels of residue between the six study fields. The final methoxyfenozide residue concentrations on litchi and longan after a 7-day preharvest interval (1.12–2.65 and 0.04–1.01 mg kg^−1^, respectively) were both below the maximum residue limit (MRL) value established in China (5 and 2 mg kg^−1^, respectively) (China Pesticide Information Network n.d.).

**Table 2:** Terminal residue of methoxyfenozide in litchi and longan

Matrix	Dosage (mg kg^−1^)	Application times	Pre-harvest intervals^a^ (PHI)/days	Average residues (mg kg^−1^)	STMR^b^ (mg kg^−1^)	Residues (mg kg^−1^)
Litchi	96	2	5	2.09	2.15	1.22–2.82
			7	1.2	1.14	0.78–1.82
			10	0.54	0.53	0.21–1.01
		3	5	2.33	2.3	1.49–3.21
			7	1.4	1.37	0.96–1.99
			10	0.76	0.74	0.36–1.23
	144	2	5	2.98	2.92	2.03–4.02
			7	1.84	1.82	1.52–2.41
			10	0.93	0.88	0.37–1.61
		3	5	3.26	3.19	2.03–4.88
			7	2.06	1.95	1.59–2.65
			10	1.21	1.15	0.59–1.85
Longan	96	2	5	0.44	0.30	0.07–1.03
			7	0.23	0.16	0.03–0.54
			10	0.11	0.10	< 0.01–0.24
		3	5	0.51	0.41	0.08–1.15
			7	0.29	0.25	0.04–0.62
			10	0.16	0.15	0.01–0.33
	144	2	5	0.64	0.59	0.13–1.23
			7	0.34	0.32	0.02–0.67
			10	0.21	0.20	0.03–0.41
		3	5	0.93	0.68	0.16–2.39
			7	0.40	0.30	0.05–1.01
			10	0.24	0.24	0.02–0.48

^a^Mean pre-harvest interval

^b^Mean supervised trials median residue

The residues in the entire litchi fruit were markedly increased compared to the pulp. It is hypothesized that the peel has a higher concentration of most residues. No direct pesticide exposure was observed in the pulp when methoxyfenozide SC was applied to the peel. Consequently, it is expected that the peel has an enhanced pesticide absorption ability. Therefore, the residue concentration that infiltrated the pulp was notably low. Per the literature, the quantity of residues of most pesticides is generally higher in the whole fruit than in the pulp (Li et al. 2017; Huang et al. 2010). However, it was observed that pesticide residues, such as methomyl, carbendazim, and thiabendazole, were substantially greater in the pulp in comparison with other parts of the fruit. Pesticide residues in fruits can be divided into two types based on their presence: the first involves pesticide adhesion to the fruit’s surface, and the second involves those that are present in the plant’s internal circulation, being distributed in various parts. Generally, the fruit’s surface is coated with wax, exhibiting crucial hydrophobic characteristics that efficiently inhibit the entrance of hydrophilic contaminants inside the fruit. In fruits, the pesticide residues primarily come from branches and leaves translocation, resulting in their accumulation within the fruit after entrance via the stem. Various factors, such as the pesticide properties, application approaches, surroundings, fruit properties, and agronomic measures, influence the pesticide distribution in fruits (Huang et al. 2010).

### Health risk analysis

Subsequently, with the help of *ADI%*, a health risk analysis was conducted to elucidate the long-term dietary risks of methoxyfenozide use in litchi and longan (Table 3). The highest *ADI%* values for methoxyfenozide for litchi and longan were 0.0055–0.033%, respectively, in line with an acceptable pesticide contamination risk level. Since the application of methoxyfenozide on litchi and longan is increasing, it is crucial to expend the research and highlight the risks linked with chronic exposure in the vulnerable age groups. The accuracy of the risk assessment data might have been influenced by the synergistic interactions of pesticides, toxicologic effects, processing variables (dried litchi and longan products), etc. Therefore, improved exposure analysis systems that can more accurately model the real world are required to limit the potential for any under- or over-estimation of dietary intake risk levels.

**Table 3:** Chronicdietary risk assessment of methoxyfenozide on litchi and longan for consumer groups

Crops	ADI (mg kg^−1^ bw)	STMR (mg kg^−1^)	*ADI%*					
			2–4 years		18–30 years		60–70 years	
			Male	Female	Male	Female	Male	Female
Litchi	0.1	0.01	0.0310	0.0331	0.0069	0.0101	0.0055	0.0064
Longan		0.01	0.0310	0.0331	0.0069	0.0101	0.0055	0.0064

## Conclusion

As China is the largest global litchi and longan-producing nation, it is necessary to elucidate the dietary risk and degradation behaviors of methoxyfenozide. In this research, a sensitive, reliable, and rapidly modified QuEChERS HPLC–MS/MS method was established and validated to detect the effects of methoxyfenozide on litchi and longan samples. The terminal residue characteristics and dissipation analyses of methoxyfenozide formulation indicate that its application does not cause increased accumulation of residues on litchi and longan. Furthermore, the health risk analysis further suggested that it is important to consider the possible chronic health risks linked with methoxyfenozide exposure in children despite the acceptable dietary risks. Based on the aforementioned data, future work should elucidate the effect of processing factors on methoxyfenozide residues in litchi and longan. Additionally, the potential hazards associated with the use of seeds and peels in phenolic extract production should also be explored.

### Supplementary Information

Below is the link to the electronic supplementary material.

Supplementary file1 (DOCX 52 KB)

Supplementary file2 (DOCX 23 KB)

## Data Availability

Data are contained within the article.

## References

[CR1] Bi YY, Yao W, Han LJ, Qiao CK, Song SY, Qin FY, Dong Q, Hao XH, Xu YJ (2021). Method validation and residue analysis of methoxyfenozide and metaflumizone in Chinese broccoli under field conditions by liquid chromatography with tandem mass spectrometry. J Sep Sci.

[CR2] Chen BX, Dong YZ, Li WJ, Quan LF, Yao Q, Xu S, Chi YY (2020). Research progress in control technique of main litchi pests and construction of IPM system. Guangdong Agricultural Sciences.

[CR3] Chen YJ, Liu XG, Dong FS, Xu J, Wu XH, Zheng YQ (2021). Characterization of the fate and distribution of methoxyfenozide in a water-plant-fish-sediment microcosm using a multimedia fugacity model. Sci Total Environ.

[CR4] China Pesticide Information Network (n.d.) http://www.Chinapesticide.org.cn/hysj/index.jhtml (2023-11-20)

[CR5] Feng YZ, Zhang GF, Zhang AJ, Zhou L, Bian YL, Pan JJ, Yang SM, Han JF, Ma XG, Qi XX, Liang L, Zuo BJ (2022). Dissipation, residue, and dietary risk assessment of methoxyfenozide, chlorantraniliprole, indoxacarb, lufenuron, and chlorfenapyr in spinach using a modified QuEChERS method combined with a tandem mass dpectrometry Technique. Agronomy.

[CR6] Fisher A, Colman C, Hoffmann C, Fritz B, Rangel J (2018). The effects of the insect growth regulators methoxyfenozide and pyriproxyfen and the acaricide bifenazate on honey bee (Hymenoptera: Apidae) forager survival. J Econ Entomol.

[CR7] Hao J, Dong LH, Chi JW, Ma YX, Zhou QY, Zhang RF, Bai YJ (2021). Health effect of longan and its present situation and prospect of healthy food development. Modern Food Sci Technol.

[CR8] Hu XX (2005). China’s pesticide industry has a long way to go: achievements and development. Chinese Pesticides.

[CR9] Huang YN, Zhang SL, Fang JB, Wu SY, Wu FK (2010). Study on residue trends of imidacloprid in pear fruit. Journal of Fruit Science.

[CR10] Li J, Jiang Y (2007). Litchi flavonoids: isolation, identification and biological activity. Molecules.

[CR11] Li Z, Zang XP, Ge Y, Wang JS, Lin XE, Ma WH, Li XG (2017). Effects of fruit bagging on residual of thiophanate-methyl and imidacloprid in mango fruit. Chinese J Trop Crops.

[CR12] Li WJ, Dong YZ, Yao Q, Chen BX (2018). Research progress in the litchi fruit borer, *Conopomorpha sinensis* (Lepidoptera:Gracillariidae). Acta Entomol Sin.

[CR13] Li YL, Yan ZB, Yin XM, Lei ZS, Zhou Z (2022). Dynamics of ecdysone titer and the interference of methoxyfenozide on diapause during the development of *Carposina sasakii* (Lepidoptera: Carposinidae). Acta Entomol Sin.

[CR14] Lin ZF, Li SS, Zhang DL, Liu SX, Li YB, Lin GZ, Chen MD (1988). The changes of oxidation and peroxidation in postharvest litchi fruit. Acta Bot Sin.

[CR15] Lin HT, Chen SJ, Xi YF, Guo SZ (2002). Observation on pericarp ultrastructure by scanning electron microscope and its relation to keeping quality of longan fruit. Trans CSAE.

[CR16] Liu WG, Su YZ, Liu J, Zhang K, Wang XY, Chen YG, Duan LC, Feng S (2021). Determination of cyflufenamid residues in 12 foodstuffs by QuEChERS-HPLC-MS/MS. Food Chem.

[CR17] Liu XJ, Yu RX, Wu SG, An XH, Lv L, Wang FD, Zhao Y, Zhao XP. (2022) Acute and chronic toxic effects of methoxyfenozide on silkworm and its ecological risk assessment. Hu XY. Study on postharvest physiology and room temperature preservation of litchi. South China University of Technology. 20014 Asian J Ecotoxicol **17**(4): 554-562.

[CR18] Pan XC, Xie BG (1996). Observation of peel structure of litchi fruit by SEM. Acta Horticulturae Sinica.

[CR19] Pan JQ, Liu HC, Liu GN, Hu YL, Chen LX, Qiu ZQ (1999). A study in blood sugar reducing, blood lipid controlling and antioxidant activities in litchi seed. Guangdong Pharm J.

[CR20] Pico Y, Farre M, Soler C, Barcelo D (2017). Identification of unknown pesticides in fruits using ultra-performance liquid chromatography-quadrupole time-of-flight mass spectrometry. Imazalil as a case study of quantification. J Chrom A.

[CR21] Rangkadilok N, Sitthimonchai S, Worasuttayangkurn L, Mahidol C, Ruchirawat M, Satayavivad J (2007). Evaluation of free radical scavenging and antityrosinase activities of standardized longan fruit extract. Food Chem Toxicol.

[CR22] Sun J (2006). Analysis of composition and antioxidant activities of litchi pericarp polysaccharide. Sci Technol Cereals.

[CR23] Sun HZ, Zhou L, Zhang XZ, Luo FJ, Yang M, Wang XR, Lou ZY, Chen ZM (2019). Residue dissipation and dietary exposure risk assessment of methoxyfenozide in cauliflower and tea via modified QuEChERS using UPLC/MS/MS. J Sci Food Agric.

[CR24] Sun JH, Cao LL, Li HL, Wang G, Wang SJ, Li F, Wang JB, Zhang L (2019). Scanning electron microscope observation on the changes of litchi exocarp inoculated with phytophthora litchi. Chinese J Trop Crops.

[CR25] Tang HX, Ma L, Huang JQ, Li YB, Liu ZH, Meng DY, Wen GY, Dong MF, Wang WM, Zhao L (2021). Residue behavior and dietary risk assessment of six pesticides in pak choi using QuEChERS method coupled with UPLC-MS/MS. Ecotoxicol Environ Saf.

[CR26] Ucles S, Belmonte N, Mezcua M, Martinez AB, Martinez-Bueno MJ, Gamon M, Fernández-Alba AR (2014). Validation of a multiclass multiresidue method and monitoring results for 210 pesticides in fruits and vegetables by gas chromatography-triple quadrupole mass spectrometry. J Environ Sci Health B.

[CR27] USAD (n.d.). http://www.nal.usda.Gov/fnic/foodcomp/cgi-bin/list_nut_edit.pl. 20 Nov 2023

[CR28] Wang Y, Wang HC, Zhou ZK, Chen HB, Hu GB, Huang XM (2009). An overview of research on litchi functional role and its active substances. J Fruit Sci.

[CR29] Wang S, Hu ZT, Zhang SF (2021). Field efficacy trial of 26% methoxyl·indoxacarb SC against *Cnaphalocrocis medinalis*. Plant Protect Sci.

[CR30] Wang WT, Cho YJ, Song JW, Kim YJ, Seo JS, Kim JH (2022). Residue behavior of methoxyfenozide and pymetrozine in Chinese cabbage and their health risk assessment. Foods.

[CR31] Wu XL, Xu ML, Zhu WY (2022). Experimental study on 70% chlorantraniliprole·methoxyfenozide WG to control the *Cnaphalocrocis medinalis* guenee. Anhui Agri Sci Bull.

[CR32] Xiao LY, Hong HJ, Pan JQ, Lv JH, Zhang SP (2007). Anti-tumor effect of semen litchi and its effect on telomerase activation of hepatoma tissue. China Pharmacy.

[CR33] Xu ZH, Li JK (2015). Research progress on diamide insect growth regulators. Jiangsu Agric Sci.

[CR34] Yao Q, Quan LF, Xu S, Dong YZ, Li WJ, Chen BX (2021). Biology and management of the litchi stink bug, Tessaratoma papillosa (Hemiptera: Tessaratomidae): progress and prospects. Acta Entomol Sin.

[CR35] Zhang WN, Liu XY, Lai Q, Xiao HJ (2021). Expression analysis of cuticular protein genes *CP22* and *CP14* in cotton bollworm *Helicoverpa armigera* and their response to the sublethal dose of methoxyfenozide. J Plant Prot.

[CR36] Zhu RX (2011) Preliminary study of storage and disease resistance to peronophythora litchi of main species of litchi fruits in Fujian. Fujian Agriculture and Forest University. 2011:49-54

