# Supplementary material for: Environmental fate and safety analysis of methoxyfenozide application to control litchi and longan pests
Source: Environ Sci Pollut Res Int. 2024 May 21;31(25):37316–25. doi: 10.1007/s11356-024-33677-0 (PMC11182796; doi:10.1007/s11356-024-33677-0)
Supplement: Supplementary file 1 — Supplementary file1 (DOCX 52 KB) [file 11356_2024_33677_MOESM1_ESM.docx]

**FIGURE CAPTIONS**

**Figure S1.** Field trials map of methoxyfenozide application in litchi (The field trials map of longan is the same as that of litchi)


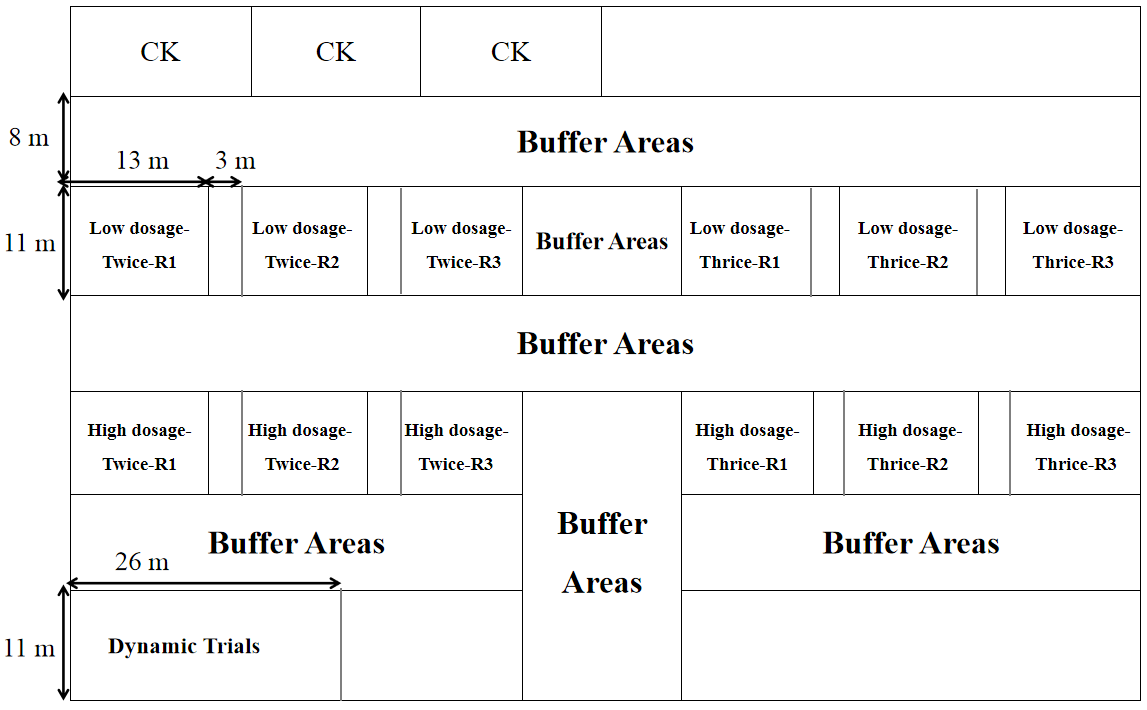


Figure S1
