# Supplementary material for: Environmental fate and safety analysis of methoxyfenozide application to control litchi and longan pests
Source: Environ Sci Pollut Res Int. 2024 May 21;31(25):37316–25. doi: 10.1007/s11356-024-33677-0 (PMC11182796; doi:10.1007/s11356-024-33677-0)
Supplement: Supplementary file 2 — Supplementary file2 (DOCX 23 KB) [file 11356_2024_33677_MOESM2_ESM.docx]

**Table S1 Previous applications of other pesticides in litchi and longan**

| No. | Crop | Trials plot | Previous applications of other pesticides |
| --- | --- | --- | --- |
| 1 | litchi | Guangzhou, Guangdong | ①Triazophos and Lambda-cyhalothrin, a 13% EC was sprayed at 75 mg a.i.·kg^-1^ doses on May 1^st^ and used twice;  ②Mancozeb, a 80% WP was sprayed at 1333 mg a.i.·kg^-1^ doses on May 9^th^ and used twice. |
| 2 |  | Maoming, Guangdong | ①Carbendazim and Mancozeb, a 62% WP was sprayed at 500 mg a.i.·kg^-1^ doses on May 10^th^, and used twice;  ②Diflubenzuron, a 40% SC was sprayed at 133.33 mg a.i.·kg^-1^ doses on May 9^th^, and used twice;  ③Gibberellic acid A4, a 40% SP was sprayed at 30 mg a.i.·kg^-1^ doses on May 15^th^, and used once. |
| 3 |  | Nanning,  Guangxi | ①Difenoconazole and Azoxystrobin, a 325 g/L SC was sprayed at 200 mg a.i.·kg^-1^ doses on June 5^th^, and used twice;  ②Lambda-cyhalothrin, a 25 g/L EC was sprayed at 25 mg a.i.·kg^-1^ doses on May 25^th^, and used twice. |
| 4 |  | Haikou,  Hainan | ①Carbendazim and Mancozeb, a 62% WP was sprayed at 500 mg a.i.·kg^-1^ doses on April 10^th^, and used twice;  ②Trichlorfon, a 80% SP was sprayed at 1142.9 mg a.i.·kg^-1^ doses on April 7^th^, and used twice. |
| 5 |  | Putian,  Fujian | ①Lufenuron and Beta-cypermethrin, a 8% EC was sprayed at 25 mg a.i.·kg^-1^ doses on June 10^th^;  ②Dimethomorph and Pyraclostrobin, a 18.7 WG was sprayed at 120 mg a.i.·kg^-1^ doses on June 5^th^. |
| 6 |  | Baoshan,  Yunnan | ①Beta-cypermethrin, a 4.5% EC was sprayed at 25 mg a.i.·kg^-1^ doses on May 10^th^, and used twice;  ②Lufenuron and Beta-cypermethrin, a 8% EC was sprayed at 25 mg a.i.·kg^-1^ doses on on April 10^th^, and used twice;  ③Kasugamycin and Copper oxychloride, a 47% WP was sprayed at 33.3 mg a.i.·kg^-1^ doses on May 1^st^, and used twice;  ④Mancozeb, a 80% WP was sprayed at 1333 mg a.i.·kg^-1^ doses on May 15^th^ and used twice. |
| 1 | longan | Guangzhou, Guangdong | ①Chlorpyrifos and Cypermethrin, a 55% EC was sprayed at 225 mg a.i.·kg^-1^ doses on June 15^th^ and used thrice.  ②Prochloraz, a 25% EC was sprayed at 225 mg a.i.·kg-1 doses on June 10th and used thrice. |
| 2 |  | Maoming, Guangdong | ①Chlorpyrifos and Cypermethrin, a 55% EC was sprayed at 225 mg a.i.·kg^-1^ doses on May 15^th^ and used twice.  ②Prochloraz, a 25% EC was sprayed at 225 mg a.i.·kg-1 doses on May 10th and used once.  ③Difenoconazole and Azoxystrobin, a 325 g/L SC was sprayed at 200 mg a.i.·kg-1 doses on May 17 th, and used twice. |
| 3 |  | Nanning,  Guangxi | ①Chlorpyrifos and Cypermethrin, a 55% EC was sprayed at 225 mg a.i.·kg^-1^ doses on June 15^th^ and used twice.  ②Mancozeb, a 80% WP was sprayed at 1333 mg a.i.·kg^-1^ doses on June 25^th^ and used twice. |
| 4 |  | Danzhou,  Hainan | ①Beta-cypermethrin, a 4.5% EC was sprayed at 25 mg a.i.·kg^-1^ doses on July 1^st^, and used twice;  ②Triazophos and Lambda-cyhalothrin, a 13% EC was sprayed at 75 mg a.i.·kg^-1^ doses on June 10^th^ and used twice;  ③Prochloraz, a 25% EC was sprayed at 225 mg a.i.·kg-1 doses on June 14th and used once. |
| 5 |  | Quanzhou,  Fujian | ①Chlorpyrifos and Cypermethrin, a 522.5 g/L EC was sprayed at 225 mg a.i.·kg^-1^ doses on July 7^th^ and used twice.  ②Mancozeb, a 80% WP was sprayed at 1333 mg a.i.·kg^-1^ doses on June 25^th^ and used twice. |
| 6 |  | Yuxi,  Yunnan | ①Chlorpyrifos and Cypermethrin, a 25% EC was sprayed at 225 mg a.i.·kg^-1^ doses on May 10^th^ and used thrice.  ②Prochloraz, a 25% EC was sprayed at 225 mg a.i.·kg-1 doses on May 14th and used twice. |

**Table S2** Average body weight and fruit intake of different age/sex consumer groups in China

| Age | Sex | Body weight (kg) | F_a_  (kg/d) | LP_b_  (kg/d) |
| --- | --- | --- | --- | --- |
| 2–4 | Male | 14.1 | 0.0437 | 0.3394 |
|  | Female | 13.4 | 0.0444 | 0.3394 |
| 18–30 | Male | 60.5 | 0.0418 | 0.5102 |
|  | Female | 52.6 | 0.0529 | 0.5102 |
| 60–70 | Male | 61.3 | 0.0338 | 0.5102 |
|  | Female | 54.3 | 0.0348 | 0.5102 |

a represents dietary consumption of fruits in different groups, b represents the large portion of fruit consumption in different groups.

**Table S3 Meteorological information during the trial period**

| No. | Crop | Trials plot | Temperature/℃ | Rainfall/  mm | Wind level |
| --- | --- | --- | --- | --- | --- |
| 1 | litchi | Guangzhou, Guangdong | 23.7～35.0 | 276.8 | 1 |
| 2 |  | Maoming, Guangdong | 23.6～36.2 | 165.1 | 1-2 |
| 3 |  | Nanning,  Guangxi | 23.1～35.5 | 256.7 | breeze |
| 4 |  | Haikou,  Hainan | 22.5～32.1 | 211.5 | 1-2 |
| 5 |  | Putian,  Fujian | 20～36 | 367.4 | 1-2 |
| 6 |  | Baoshan,  Yunnan | 16.1～31.4 | 14.2 | 1-2 |
| 1 | longan | Guangzhou, Guangdong | 23.0～38.0 | 220.4 | 1 |
| 2 |  | Maoming, Guangdong | 22.0～36.2 | 122.0 | 1-2 |
| 3 |  | Nanning,  Guangxi | 23.1～37.5 | 207.3 | breeze |
| 4 |  | Danzhou,  Hainan | 24.0～37.0 | 137.0 | slight breeze |
| 5 |  | Quanzhou,  Fujian | 22.8～37.0 | 42.6 | 1-2 |
| 6 |  | Yuxi,  Yunnan | 13.0～27.2 | 177.5 | 1-2 |
